# Dual-layer spectral-detector CT for predicting microsatellite instability status and prognosis in locally advanced gastric cancer

**DOI:** 10.1186/s13244-023-01490-x

**Published:** 2023-09-19

**Authors:** Yongjian Zhu, Peng Wang, Bingzhi Wang, Zhichao Jiang, Ying Li, Jun Jiang, Yuxin Zhong, Liyan Xue, Liming Jiang

**Affiliations:** 1 Department of Diagnostic Radiology, National Cancer Center/National Clinical Research Center for Cancer/Cancer Hospital, Chinese Academy of Medical Sciences and Peking Union Medical College, Beijing, 100021 China; 2 Department of Pathology, National Cancer Center/National Clinical Research Center for Cancer/Cancer Hospital, Chinese Academy of Medical Sciences and Peking Union Medical College, Beijing, 100021 China; 3 Department of Medical Oncology, National Cancer Center/National Clinical Research Center for Cancer/Cancer Hospital, Chinese Academy of Medical Sciences and Peking Union Medical College, Beijing, 100021 China; 4 Department of Pancreatic and Gastric Surgery, National Cancer Center/National Clinical Research Center for Cancer/Cancer Hospital, Chinese Academy of Medical Sciences and Peking Union Medical College, Beijing, 100021 China

**Keywords:** Gastric cancer, Dual-layer spectral detector CT, Microsatellite instability, Quantitative parameters, Nomogram

## Abstract

**Objective:**

To construct and validate a prediction model based on dual-layer detector spectral CT (DLCT) and clinico-radiologic features to predict the microsatellite instability (MSI) status of gastric cancer (GC) and to explore the relationship between the prediction results and patient prognosis.

**Methods:**

A total of 264 GC patients who underwent preoperative DLCT examination were randomly allocated into the training set (*n* = 187) and validation set (*n* = 80). Clinico-radiologic features and DLCT parameters were used to build the clinical and DLCT model through multivariate logistic regression analysis. A combined DLCT parameter (C_DLCT_) was constructed to predict MSI. A combined prediction model was constructed using multivariate logistic regression analysis by integrating the significant clinico-radiologic features and C_DLCT_. The Kaplan–Meier survival analysis was used to explore the prognostic significant of the prediction results of the combined model.

**Results:**

In this study, there were 70 (26.52%) MSI-high (MSI-H) GC patients. Tumor location and CT_N staging were independent risk factors for MSI-H. In the validation set, the area under the curve (AUC) of the clinical model and DLCT model for predicting MSI status was 0.721 and 0.837, respectively. The combined model achieved a high prediction efficacy in the validation set, with AUC, sensitivity, and specificity of 0.879, 78.95%, and 75.4%, respectively. Survival analysis demonstrated that the combined model could stratify GC patients according to recurrence-free survival (*p* = 0.010).

**Conclusion:**

The combined model provides an efficient tool for predicting the MSI status of GC noninvasively and tumor recurrence risk stratification after surgery.

**Critical relevance statement:**

MSI is an important molecular subtype in gastric cancer (GC). But MSI can only be evaluated using biopsy or postoperative tumor tissues. Our study developed a combined model based on DLCT which could effectively predict MSI preoperatively. Our result also showed that the combined model could stratify patients according to recurrence-free survival. It may be valuable for clinicians in choosing appropriate treatment strategies to avoid tumor recurrence and predicting clinical prognosis in GC.

**Key points:**

• Tumor location and CT_N staging were independent predictors for MSI-H in GC.

• Quantitative DLCT parameters showed potential in predicting MSI status in GC.

• The combined model integrating clinico-radiologic features and C_DLCT_ could improve the predictive performance.

• The prediction results could stratify the risk of tumor recurrence after surgery.

**Graphical Abstract:**

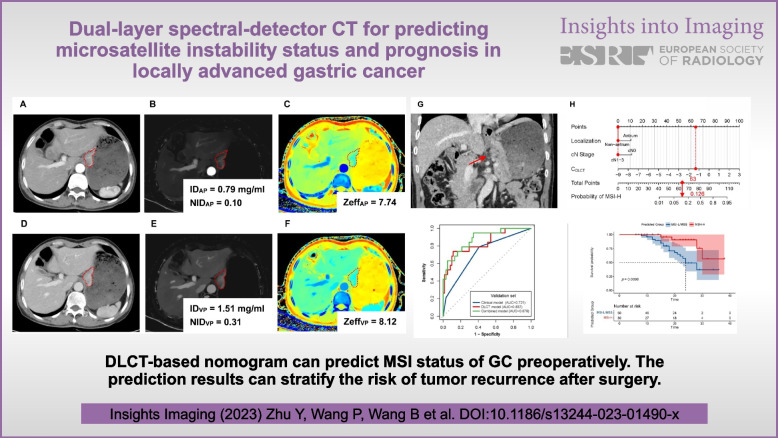

**Supplementary Information:**

The online version contains supplementary material available at 10.1186/s13244-023-01490-x.

## Introduction

Gastric cancer (GC) is one of the most common malignant tumors in the world. Its incidence and mortality rank fifth and fourth among malignant tumors globally in 2020 [1], respectively. Approximately 40% of newly diagnosed gastric cancer cases occurred in China, but the overall 5-year survival rate was only about 35% [2, 3]. Microsatellite instability (MSI) phenotype GC is a special molecular subtype based on molecular markers and next-generation sequencing [4, 5].

MSI is essentially caused by deficient DNA mismatch repair machinery, resulting in a high mutation phenotype, CpG island methylation, and *MLH1* gene silence [6]. According to MSI status, GC can be classified into MSI-high (MSI-H), MSI-low (MSI-L), or microsatellite stable (MSS) [7]. The prevalence of MSI-H GC is reported to be about 5.6 to 33.3% [8]. MSI-H is one of the favorable prognostic factors for stages I to III GC [6–8]. Some randomized clinical trials have confirmed that MSI-H GC patients could benefit from immunotherapy [6, 9, 10], but were resistant to traditional chemotherapy [11]. Besides, MSI status evaluation is also one of the screening methods for Lynch syndrome [6, 7]. As a result, an accurate assessment of the MSI status of GC has substantial therapeutic and prognostic significance. The NCCN and ESMO guidelines for gastric cancer both recommend routine MSI testing for all GC patients [12, 13].

Currently, MSI testing relies on pathological examination [6, 14]. However, the detection techniques are complicated and expensive. Tissue specimens cannot be obtained for testing from patients who are unsuitable for endoscopic examination or surgery. In addition, due to the intratumoral and temporal heterogeneity of GC, biopsy specimens often fail to reflect the features of the entire tumor accurately [15, 16]. As a result, there is an urgent need to explore non-invasive, simple, and practical methods for determining the MSI status of GC preoperatively.

CT imaging examination is one of the important methods for preoperative evaluation of GC [12, 13]. Dual-layer spectral detector CT (DLCT) is the latest generation of energy CT technology, which could obtain many quantitative spectral CT parameters [17]. Since the separation of spectral data occurs at the detector level and spectral data are available during every scan, DLCT requires no preplanning and preselection of dual-energy scanning mode, in contrast to dual-source and rapid voltage-switching systems [17, 18]. Another advantage of DLCT is that it does not increase the radiation doses of patients compared with source-based imaging technologies [18]. Previous studies have demonstrated that spectral CT could be utilized to improve the image quality and analyze the pathological features of various tumors quantitatively [19–22].

However, no studies using DLCT to evaluate the MSI status of GC have been reported. Therefore, the purpose of this study was to investigate the predictive value of DLCT parameters for MSI status in GC and construct and validate a combined prediction model based on DLCT quantitative parameters and clinico-radiologic features. The stratification ability of the combined model for the prognosis of GC patients will also be explored to provide a basis for clinically individualized and accurate treatment.

## Materials and methods

### Patients

This retrospective study was approved by the Institutional Ethics Committee of the Cancer Hospital, Chinese Academy of Medical Sciences (No. 20/412–2608), and waived the requirement of informed consent from patients.

**Fig. 1:**
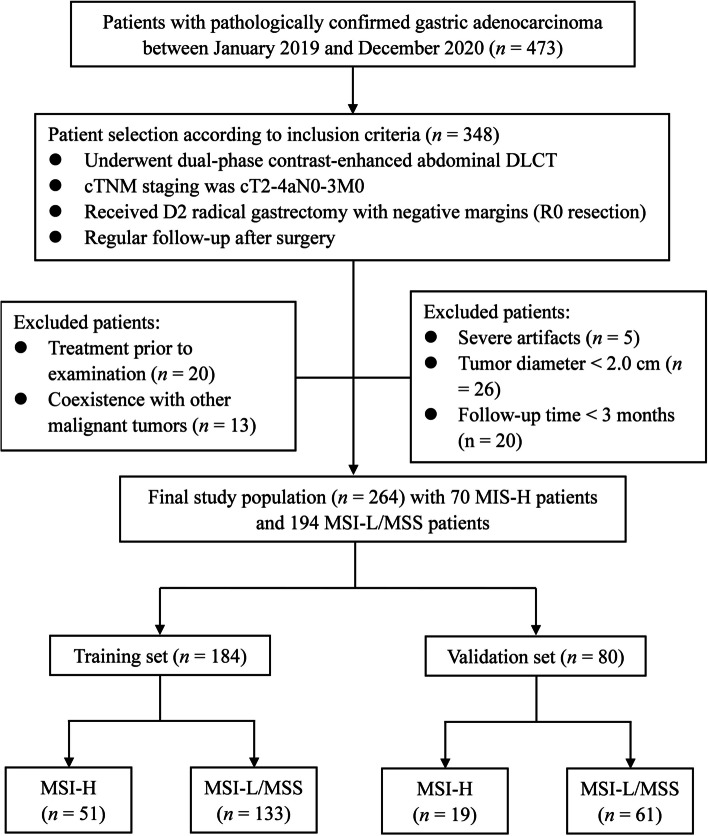
Flowchart of the recruitment of the study population. *DLCT* dual-layer spectral detector CT; *MSI-H* microsatellite instability high; *MSI-L/MSS* microsatellite instability low or microsatellite stable

The consecutive patients with pathologically confirmed gastric adenocarcinoma in our institution between January 2019 and December 2020 were initially collected. The inclusion criteria were as follows: (1) underwent dual-phase contrast-enhanced abdominal DLCT examination, (2) clinical TNM staging was cT2-4aN0-3M0, (3) received D2 radical gastrectomy within 30 days after DLCT examination, and (4) regular follow-up after surgery. The exclusion criteria were as follows: (1) received local or systematic treatment before examination, (2) coexistence with other malignant tumors, (3) severe respiratory or gastrointestinal movement artifacts, (4) maximum tumor diameter < 2.0 cm, and (5) follow-up time less than 3 months after surgery. Finally, a total of 264 patients were enrolled in this study, including 187 men and 77 women with a median age of 60 years (range, 30–75). The patients were randomly divided into a training set (*n* = 184) and a validation set (*n* = 80) at a ratio of 7:3 using the random seed method. The flow chart of the study population is displayed in Fig. 1.

### DLCT examination

The examination was performed using a DLCT scanner (IQon Spectral CT, Philips Healthcare, Best, The Netherlands). The scanning protocol includes nonenhanced, arterial phase (AP), and venous phase (VP) scans, which cover the whole abdomen and pelvis. Conventional CT images and spectral base images were generated for subsequent analysis. Detailed information on CT scanning protocol and imaging reconstruction are listed in Additional file 1: Text S1.

### Image analysis

Image analysis was performed by two radiologists (Y.J.Z. and Y.L., with 10 and 20 years of experience in gastrointestinal imaging, respectively).

For the DLCT quantitative parameter, the spectral data of AP and VP were transferred to the workstation (IntelliSpace Portal version 9.0, Philips Healthcare) to obtain virtual monoenergetic images (VMI), iodine density (ID) maps, and effective atomic number (Zeff) maps. The tumor region of interest (ROI) was manually traced along the tumor contour at the slice of the largest tumor diameter in conventional CT images. Another circular ROI was placed on the descending aorta. The ROI was automatically copied to the VMI at 40 keV and 100 keV, ID map, and Zeff map to obtain quantitative parameters. The following quantitative parameters in AP and PP were measured and calculated: (1) CT values of 40 keV and 100 keV (CT_40keV_ and CT_100keV_); (2) ID values of tumors and aorta (ID_tumor_ and ID_aorta_); (3) Zeff value; (4) the normalized iodine density (NID) values: NID = ID_tumor_/ID_aorta_; (5) the slope of the spectral curve (λHU), λHU = (CT_40keV_-CT_100keV_)/60.

The following imaging features were measured and evaluated in conventional CT images: (1) maximum diameter (MD) was measured as the longest tumor diameter; (2) maximum thickness (MT) was measured as the short axis perpendicular to the MD; (3) tumor location; (4) CT evaluated T stage (CT_T stage), N stage (CT_N stage), and clinical tumor stage (cTNM stage) which was classified according to AJCC^8th^ staging system [23], with reference to the CT imaging classification described in the Chinese clinical guidelines for the diagnosis and treatment of gastric cancer [24]. A final consensus was reached by discussing with a third professor (L.M.J., with over 35 years of experience) in case of disagreement during assessment.

### Pathological evaluation

MSI testing was performed according to the recommendations for mismatch repair (MMR) and MSI Testing from the College of American Pathologist [14]. In this study, immunohistochemical staining analysis was used to detect the expression levels of four MMR proteins: MLH1, MSH2, MSH6, and PMS2. MSI status was classified as MSI-L/MSS if all four markers were positive, or MSI-H if complete loss of expression was observed in any one of the MMR proteins.

### Clinical treatment and postoperative follow-up

All patients underwent D2 radical gastrectomy. Adjuvant chemotherapy was administered according to the pathological staging and risk factors. Patients were followed up regularly at the outpatient clinic after surgery. The recurrence-free survival (RFS) time was defined as the time interval from surgery to the first date of tumor recurrence during follow-up or the last date of follow-up period without tumor recurrence. Tumor recurrence referred to local recurrence, distant metastasis, or death caused by gastric cancer as detected by imaging or pathological examination.

### Statistical analysis and model construction

Variables were compared using independent sample *t* test, Mann–Whitney *U* test, *χ*^2^, or Fisher exact test as appropriate. Interobserver variability of quantitative parameters was assessed with intraclass correlation coefficient (ICC). Interobserver agreement of subjective imaging findings was evaluated by Cohen’s kappa coefficients (*κ*-values). The interpretation of ICC and *κ*-values was defined as follows [25]: excellent (> 0.81), good (0.61–0.80), moderate (0.41–0.60), fair (0.21–0.40), and poor (< 0.20).

Multivariate logistic regression was used to identify independent predictors and construct clinical and DLCT prediction models. A combined DLCT parameter (C_DLCT_) was generated based on the linear predictors of the regression equation. A combined prediction model and a visualized nomogram were established by multivariate logistic regression using independent clinico-radiologic features and C_DLCT_. Finally, the validation set was used to assess the model generalization. The receiving operating curve (ROC) was used to evaluate the predictive performance. The Hosmer–Lemeshow goodness-of-fit test and calibration curve were used to evaluate the model’s calibration. Decision curve analysis (DCA) was used to evaluate the clinical value of the combined model. The value of the combined model in RFS risk stratification was evaluated using Kaplan–Meier survival curves and tested by log-rank test.

A two-tailed *p* < 0.05 was considered statistically significant. All statical analysis was performed using R software (version 4.2.2; R Foundation, Vienna, Austria).

## Results

### Clinico-radiologic characteristics

The clinico-radiologic characteristics of the 264 GC patients are described in Table 1. Of the 264 GC patients enrolled in this study, 26.52% (70/264) were categorized into the MSI-H group. The clinico-radiologic characteristics between the training set and validation set showed no significant difference (all *p* > 0.05) (Table 1).

**Table 1:** Clinicopathological data of gastric cancer patients in training set and validation set

Variables	Training set (*n* = 184)			Validation set (*n* = 80)			*p* value^†^
	MSI-H (*n* = 51)	MSI-L/MSS (*n* = 133)	*p* value^*^	MSI-H (*n* = 19)	MSI-L/MSS (*n* = 61)	*p* value^*^	
Age, years	63.24 ± 8.95	58.73 ± 11.20	**0.011**	63.37 ± 5.57	57.15 ± 10.70	**0.018**	0.340
Gender			0.367			0.366	0.492
Male	38	90		12	47		
Female	13	43		7	14		
CEA			0.639			1.000	0.188
Normal	44	1		15	47		
Elevated	7	22		4	14		
CA19-9			0.870			0.797	0.169
Normal	46	121		17	51		
Elevated	5	12		2	10		
MD, cm	4.43 ± 1.79	4.62 ± 1.63	0.498	4.07 ± 1.17	4.68 ± 1.72	0.154	0.860
MT, cm	2.04 ± 0.45	2.03 ± 0.45	0.931	2.23 ± 0.59	2.08 ± 0.42	0.223	0.182
Tumor location			** < 0.001**			**0.026**	0.367
Cardia/fundus	9	45		4	19		
Corpus	12	55		2	21		
Antrum/pylorus	30	33		13	21		
CT_T staging			**0.046**			**0.017**	0.443
T2	15	26		7	7		
T3	22	44		7	19		
T4a	14	63		5	35		
CT_N staging			** < 0.001**			**0.003**	0.976
N0	15	9		6	6		
N1	22	36		9	15		
N2	13	58		4	27		
N3	1	30		0	13		
cTNM staging (AJCC 8^th^)			**0.002**			**0.019**	0.525
I	8	6		5	4		
II	15	22		6	12		
III	28	105		8	45		
Histopathological type			0.187			0.105	0.972
Adenocarcinoma	36	77		12	36		
Poorly cohesive	7	38		2	18		
Mucinous	5	12		2	5		
Mixed	3	6		3	2		
Histological grade			0.468			0.149	0.716
Well	5	9		4	4		
Moderate	8	30		2	12		
Poor	38	94		13	45		
Lauren type			**0.014**			**0.021**	0.790
Intestinal type	32	52		13	20		
Mixed type	12	46		4	24		
Diffuse type	7	35		2	17		
Lymphovascular invasion			0.272			0.771	0.322
Positive	23	72		8	28		
Negative	28	61		11	33		
Perineural invasion			0.589			0.462	0.581
Positive	20	58		6	25		
Negative	31	75		13	36		

Data are expressed as mean ± SD or number with percentage in parentheses. Statistically significant results are marked in bold

*CA19-9* Carbohydrate antigen 19–9, *CEA* Carcinoembryonic antigen, *DLCT* Dual-layer spectral detector CT, *MD* Maximum diameter, *MSI-H* Microsatellite instability high, *MSI-L/MSS* Microsatellite instability low or microsatellite stable, *MT* Maximum thickness

^*^Comparison between MSI -H group and MSI-L/MSS group

^†^Comparison between training set and validation set

Age, tumor location, CT_T stage, CT_N stage, cTNM stage, and Lauren classification exhibited significant differences between the MSI-H group and the MSI-L/MSS group both in the training and validation sets (all *p* < 0.05) (Table 1).

### Interobserver agreement

The ICCs measured by two radiologists for MD, MT, and DLCT quantitative parameters were 0.867–0.946 (Additional file 1: Table S1), showing excellent interobserver agreements. The consistency of subjective imaging findings between observers was good to excellent with kappa values of 0.787–0.970 (Additional file 1: Table S2).

### Comparison of DLCT quantitative parameters between MSI-H and MSI-L/MSS GC

In both training set and validation set, the DLCT parameters in VP CT_40keV_VP_, CT_70keV_VP_, ID_VP_, NID_VP_, Zeff_VP_, and λHU_VP_ of the MSI-H group were significantly lower than those of the MSI-L/MSS group (all *p* < 0.05). No significant differences were observed in the DLCT parameters of AP between the two groups (all *p* > 0.05) (Table 2).

**Table 2:** Comparison of DLCT quantitative parameters between different MSI status in training set and validation set

Parameters	Training set (*n* = 184)			Validation set (*n* = 80)		
	MSI-H (*n* = 51)	MSI-L/MSS (*n* = 133)	*p* value^*^	MSI-H (*n* = 51)	MSI-L/MSS (*n* = 133)	*p* value^*^
Arterial phase						
CT_40keV_AP_, HU	120.07 (95.50, 150.99)	126.87 (109.36, 150.24)	0.290	115.49 (87.77, 152.19)	120.01 (86.74, 150.50)	0.950
CT_70keV_AP_, HU	65.98 (55.98, 76.40)	66.19 (59.07, 78.82)	0.698	63.43 (55.15, 74.65)	64.24 (51.33, 76.52)	0.968
ID_AP_, mg/ml	0.96 (0.70, 1.32)	0.99 (0.79, 1.42)	0.287	0.87 (0.74, 1.17)	0.91 (0.64, 1.27)	0.928
NID_AP_	0.10 (0.08, 0.14)	0.11 (0.08, 0.16)	0.517	0.09 (0.06, 0.11)	0.10 (0.08, 0.13)	0.173
Zeff_AP_	7.81 (7.67, 7.90)	7.83 (7.69, 8.03)	0.394	7.80 (7.65, 7.95)	7.80 (7.65, 8.03)	0.991
λHU_VP_	1.37 (1.02, 1.55)	1.39 (1.06, 2.02)	0.340	1.21 (0.68, 1.42)	1.25 (0.85, 1.82)	0.494
Venous phase						
CT_40keV_VP_, HU	145.38 (124.25, 193.55)	167.37 (145.80, 198.73)	0.012	144.96 (130.04, 173.92)	177.77 (150.14, 197.73)	0.006
CT_70keV_VP_, HU	79.20 (69.59, 90.48)	85.70 (73.49, 97.48)	0.035	73.82 (66.78, 85.00)	84.60 (74.21, 98.86)	0.026
ID _VP_, mg/ml	1.11 (0.98, 1.42)	1.52 (1.34, 1.87)	< 0.001	1.21 (0.98, 1.38)	1.56 (1.39, 1.99)	< 0.001
NID_VP_	0.26 (0.20, 0.29)	0.34 (0.28, 0.41)	< 0.001	0.23 (0.18, 0.29)	0.34 (0.28, 0.40)	< 0.001
Zeff_VP_	7.87 (7.81, 7.99)	8.13 (8.05, 8.27)	< 0.001	7.83 (7.75, 7.95)	8.15 (8.07, 8.35)	< 0.001
λHU_VP_	1.31 (0.90, 1.81)	2.19 (1.78, 2.92)	< 0.001	1.50 (1.18, 2.98)	2.30 (1.82, 2.97)	0.002

Data are given as median (inter-quartile ranges)

*AP* Arterial phase, *DLCT* Dual-layer spectral detector CT, *ID* Iodine density, *λHU* The slope of the spectral curve, *MSI* Microsatellite instability, *MSI-H* Microsatellite instability high, *MSI-L/MSS* Microsatellite instability low or microsatellite stable, *NID* Normalized iodine density, *VP* Venous phase, *Zeff* Effective atomic number

^*^p value represents comparison between MSI-H group and MSI-L/MSS group

### Predictive performance of DLCT quantitative parameters for MSI status

**Fig. 2:**
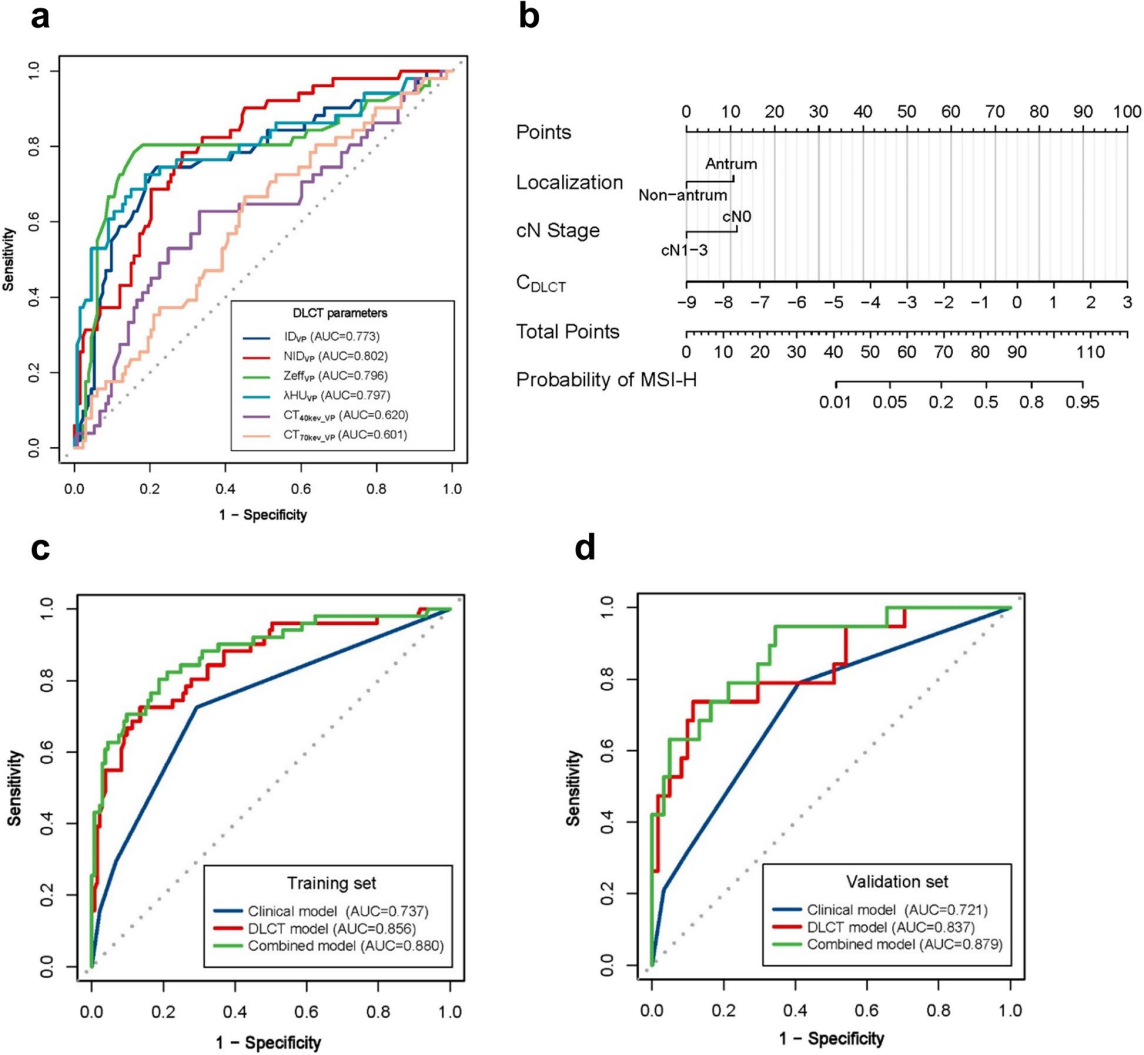
ROC curves of the DLCT parameters and prediction models, and the combined prediction nomogram. **a** Predictive performance of DLCT parameters in predicting MSI status of GC in the training set. **b** The combined nomogram for discriminating MSI status of gastric cancer in the training set. The prediction nomogram was built based on the multivariate logistic model integrated with the variables of clinical features and C_DLCT_. **c** ROC curves of the clinical, DLCT, and combined models for the prediction of microsatellite instability status in the training set. **d** ROC curves of the clinical, DLCT, and combined models for the prediction of microsatellite instability status in the training set. Both in the training set and the validation set, the combined model showed the best prediction performance. *C*_*DLCT*_ combined dual-layer spectral detector CT parameters; *DLCT* dual-layer spectral detector CT; *GC*, gastric cancer; *ID* iodine density; *λHU* the slope of the spectral curve; *MSI* microsatellite instability; *MSI-H* microsatellite instability high; *NID* normalized iodine density; *VP* venous phase; *Zeff* effective atomic number

The predictive performance of significant DLCT quantitative parameters for MSI status of GC in the training set is described in Table 3 and Fig. 2a. Among these single parameters, NID_VP_ showed the best predictive performance for discriminating MSI status, with an AUC of 0.795 (95% CI 0.709–0.882), accuracy of 82.61%, sensitivity of 78.43%, and specificity of 84.21%.

**Table 3:** Predictive efficacy of DLCT quantitative parameters for MSI status of GC in the training set

Parameters	Threshold	AUC (95% CI)	Sensitivity (%)	Specificity (%)	Accuracy (%)
CT_40keV_VP_, HU	154.30	0.620 (0.525, 0.715)	62.75 (32/51)	66.92 (89/133)	65.76 (121/184)
CT_70keV_VP_, HU	84.82	0.601 (0.510, 0.691)	66.67 (34/51)	54.89 (73/133)	58.15 (107/184)
ID_VP_, mg/ml	1.32	0.773 (0.690, 0.855)	74.51 (38/51)	78.20 (104/133)	77.17 (142/184)
NID_VP_	0.29	0.802 (0.734, 0.870)	78.43 (40/51)	71.43 (95/133)	73.37 (135/184)
Zeff_VP_	8.00	0.795 (0.709, 0.882)	78.43 (40/51)	84.21 (112/133)	82.61 (152/184)
λHU_VP_	1.66	0.797 (0.714, 0.880)	72.55 (37/51)	81.20 (108/133)	78.80 (145/184)

Except for AUC, values are percentage with number of examinations in parentheses

*AUC* Area under the curve, *CI* Confidence interval, *DLCT* Dual-layer spectral detector CT, *GC* Gastric cancer, *ID* Iodine density, *λHU* The slope of the spectral curve, *MSI* Microsatellite instability, *NID* Normalized iodine density, *VP* Venous phase, *Zeff* Effective atomic number

### Construction and validation of models for predicting MSI status in GC

Significant features were used to build the clinical and DLCT models through multivariate logistic regression, respectively. The results showed that tumor location (OR 4.343, 95% CI 2.120–8.894, *p* < 0.001) and CT_N stage (OR 5.768, 95%CI 2.200–15.121, *p* < 0.001) were independent risk factors for MSI-H (Table 4). The combined spectral CT parameters were constructed as follows: C_DLCT_ = 15.922 − 12.899 × NID_VP_ − 1.405 × Zeff_VP_ − 0.943 × λHU_VP_. The predictive performance of clinical and DLCT models is described in Table 5.

**Table 4:** Construction of clinical, DLCT, and combined models through multivariate logistic analysis

Models	β	S.E	Wald	*p* value	OR	95% CI for OR	
						Lower	Upper
Clinical							
Tumor location (antrum/pylorus)	1.468	0.366	16.117	< 0.001	4.343	2.120	8.894
CT_N Staging (N0)	1.752	0.492	12.697	< 0.001	5.768	2.200	15.121
Constant	− 1.841	0.269	46.814	< 0.001	0.159		
DLCT							
NID_VP_	− 12.899	2.925	19.448	< 0.001	2.500E − 6	8.097E − 9	0.001
Zeff_VP_	− 1.405	0.506	7.715	0.005	0.245	0.091	0.661
λHU_VP_	− 0.943	0.310	9.222	0.002	0.390	0.212	0.716
Constant	15.922	4.414	13.013	< 0.001	8.216E + 06		
Combined							
Tumor location (antrum/pylorus)	1.194	0.441	7.333	0.007	3.301	1.391	7.837
CT_N Staging (N0)	1.280	0.561	5.201	0.023	3.597	1.197	10.808
C_DLCT_	0.937	0.171	30.074	< 0.001	2.552	1.826	3.567
Constant	− 0.740	0.318	5.404	0.020	0.477		

A stepwise forward method was used to assess the best independent predictor of microsatellite instability status

*β* Coefficient, *C*_*DLCT*_ Combined DLCT parameters, *CI* Confidence interval, *DLCT* Dual-layer spectral detector CT, *λHU* The slope of the spectral curve, *NID* Normalized iodine density, *OR* Odds ratio, *S.E.* Standard error, *VP* Venous phase, *Wald* Wald chi-square, *Zeff* Effective atomic number

**Table 5:** Predictive efficacy of clinical, DLCT, and combined model for MSI status of gastric cancer

Models	AUC (95% CI)	Sensitivity (%)	Specificity (%)	Accuracy (%)
Training set				
Clinical	0.737 (0.660~0.813)	72.55 (37/51)	70.68 (94/133)	71.20 (131/184)
DLCT	0.856 (0.792~0.920)	72.55 (37/51)	86.47 (115/133)	82.61 (152/184)
Combined	0.880 (0.820~0.939)	80.39 (41/51)	81.20 (108/133)	80.98 (149/184)
Validation set				
Clinical	0.721 (0.596~0.846)	78.95 (15/19)	59.02 (36/61)	63.75 (51/80)
DLCT	0.837 (0.725~0.949)	73.68 (14/19)	88.52 (54/61)	85.00 (68/80)
Combined	0.879 (0.791~0.968)	78.95 (15/19)	75.40 (46/61)	76.25 (61/80)

*AUC* Area under the curve, *CI* Confidence interval, *DLCT* Dual-layer spectral detector CT, *MSI* Microsatellite instability

Tumor location, CT_N stage, and C_DLCT_ were input into the multivariate logistic regression to construct a combined prediction model (Table 4). A risk score (RS) for MSI-H using the linear predictor based on the regression coefficients could be calculated as follows: RS =  − 0.740 + 0.937 × C_DLCT_ + 1.194 × tumor location (antrum/pylorus) + 1.280 × CT_N stage (N0). Then, the probability of MSI-H could be calculated as follows: Probability = 1/[1 + exp(-RS)].

The visualized nomogram for predicting MSI of GC is displayed in Fig. 2b. The AUC of the combined model for predicting MSI-H were 0.880 in the training set and 0.879 in the validation set, respectively (Table 5).

The ROC curves of the three models in the training and validation sets are shown in Fig. 2c and d. The calibration curve and Hosmer–Lemeshow test (all *p* > 0.05) showed that all prediction models had good agreement of the model fit. The DCA curve demonstrated that the combined prediction model had better clinical net benefit (Additional file 1: Fig. S1).

**Fig. 3:**
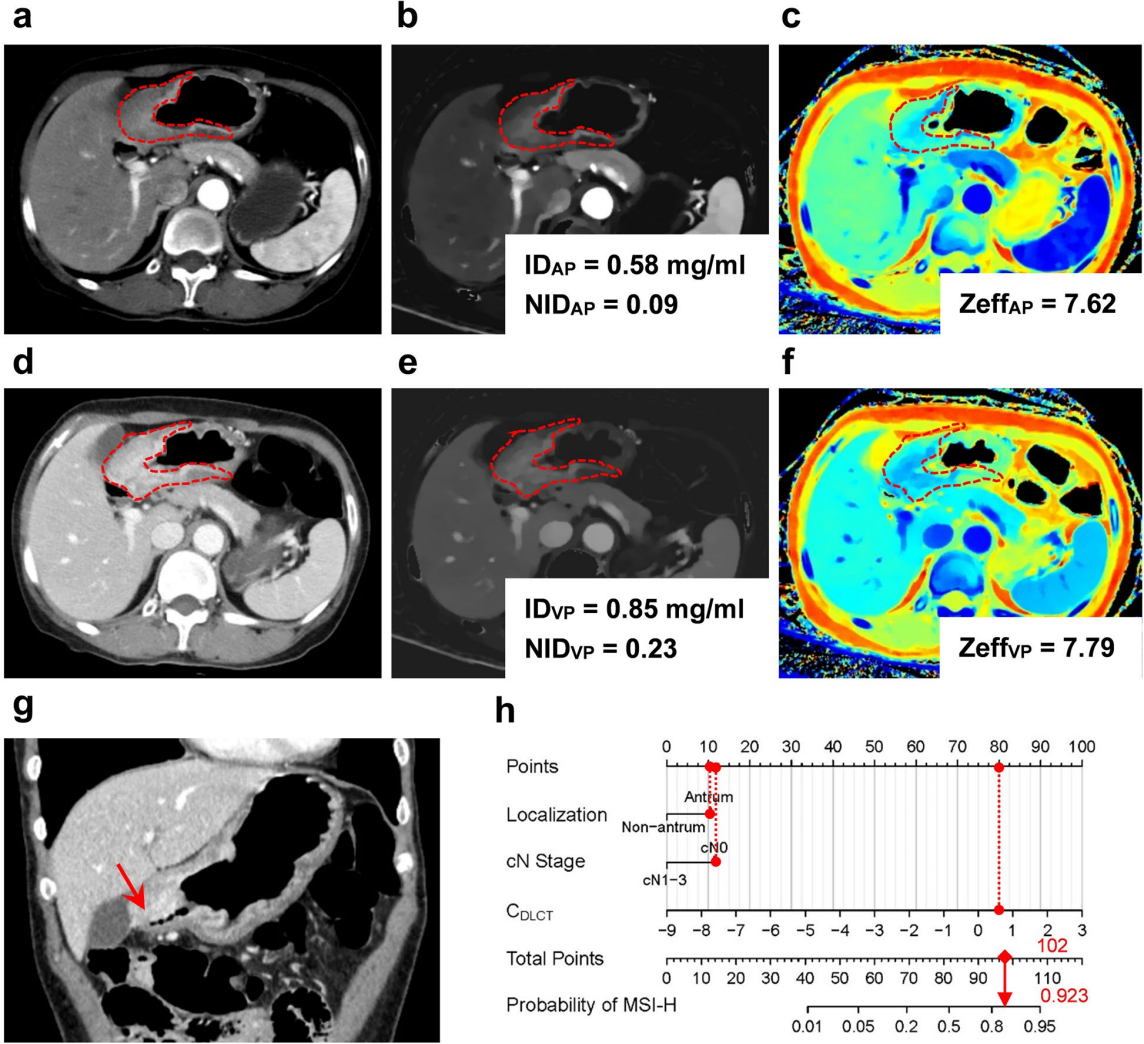
Dual-layer spectral-detector CT images of a 59-year-old male with MSI-H gastric cancer. A 64-year-old male with poorly differentiated adenocarcinoma. **a–c** The conventional CT images (**a**), ID map (**b**), and Zeff map (**c**) in arterial phase, respectively. **d–f** The conventional CT images (**d**), ID map (**e**), and Zeff map (**f**) in venous phase, respectively. Irregular thickening of the gastric wall in the antrum and local masses with soft tissue density can be seen, exhibiting progressive enhancement. The red dashed line shows the freehand mode ROIs of the tumor. ID values, NID values, and Zeff values are indicated in the figures, respectively. **g** The coronal image of venous phase. The red arrow shows the thickened wall of the gastric lower body and antrum, without suspicion of enlarged lymph nodes. **h** The utilization of the nomogram to predict the risk of MSI-H. Corresponding score of each feature is seen on points scale. When point scores for all variables were added, total scores and corresponding probability of MSI-H were presented on total points and probability scales, respectively. Moreover, observation values are superimposed on plot and are shown as red circles or diamonds and solid or dashed droplines. C_DLCT_ value of this patient was 1.21. After points for each predictor were added, total number of points was 102. Corresponding risk of MSI-H was 0.923. Histologic examination verified MSI-H status. *AP* arterial phase; *ID* iodine density; *λHU* the slope of the spectral curve; *MSI-H* microsatellite instability high; *NID* normalized iodine density; *VP* venous phase; *Zeff* effective atomic number

**Fig. 4:**
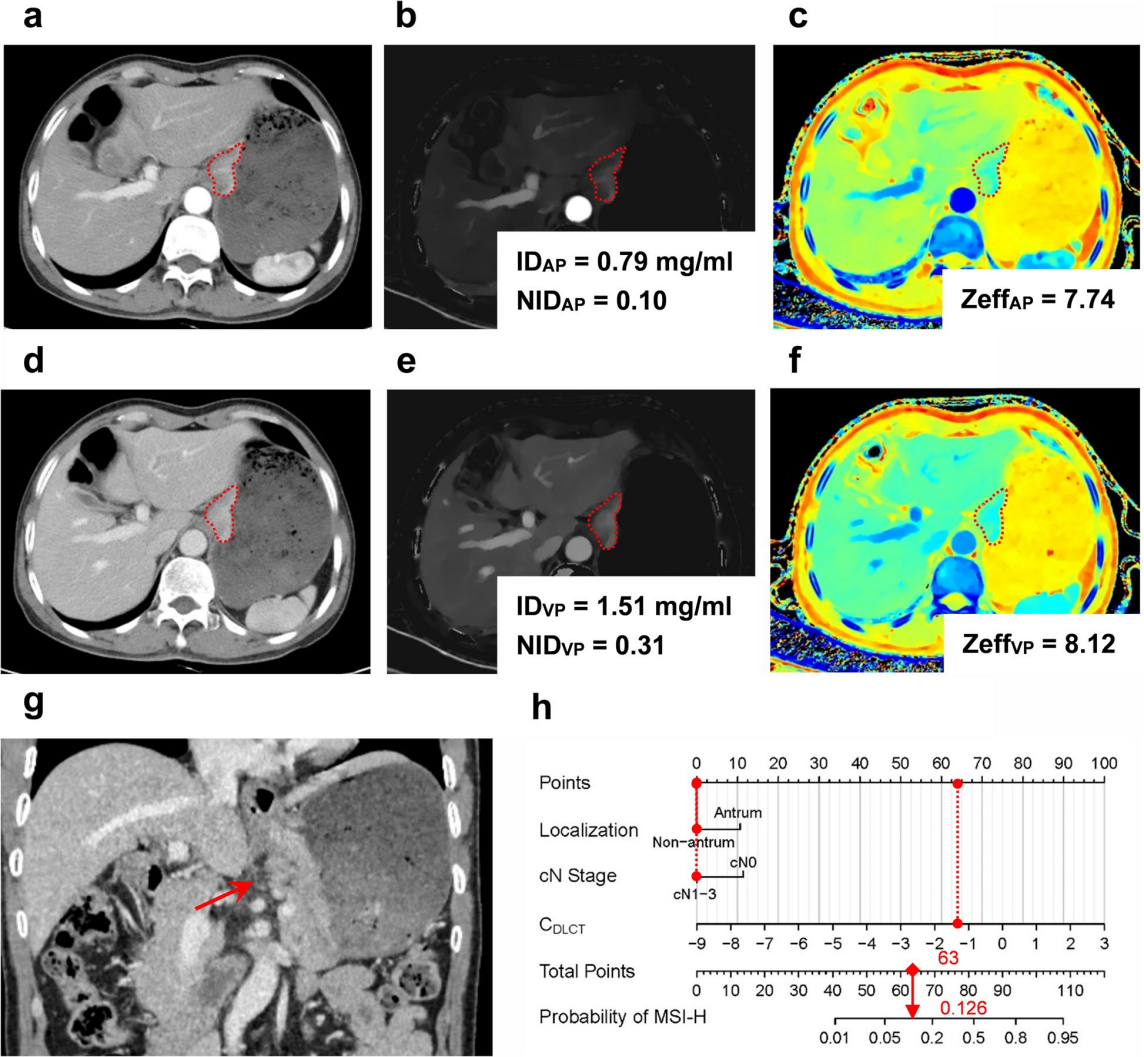
Dual-layer spectral-detector CT images of a 48-year-old male with MSI-L/MSS gastric cancer. A 48-year-old male with poorly differentiated adenocarcinoma. **a–c** The conventional CT images (**a**), ID map (**b**), and Zeff map (**c**) in arterial phase, respectively. **d–f** The conventional CT images (**d**), ID map (**e**), and Zeff map (**f**) in venous phase, respectively. Gastric wall of cardia and upper corpus was irregularly thickened. The red dashed line shows the freehand mode ROIs of the tumor. ID values, NID values, and Zeff values are indicated in the figures, respectively. **g** The coronal image of venous phase. The red arrow shows the thickened wall of the gastric cardia and upper corpus, with suspicion metastatic lymph node. **h** The utilization of the nomogram to predict the status of MSI. Corresponding score of each feature is seen on points scale. When point scores for all variables were added, total scores and corresponding probability of MSI-H were presented on total points and probability scales, respectively. Moreover, observation values are superimposed on plot and are shown as red circles or diamonds and solid or dashed droplines. C_DLCT_ value of this patient was − 1.27. After points for each predictor were added, total number of points was 63. Corresponding risk of MSI-H was 0.126. Histologic examination verified MSI-H status. *AP* arterial phase; *ID* iodine density; *λHU* the slope of the spectral curve; *MSI-H* microsatellite instability high; *NID* normalized iodine density; *VP* venous phase; *Zeff* effective atomic number

**Fig. 5:**
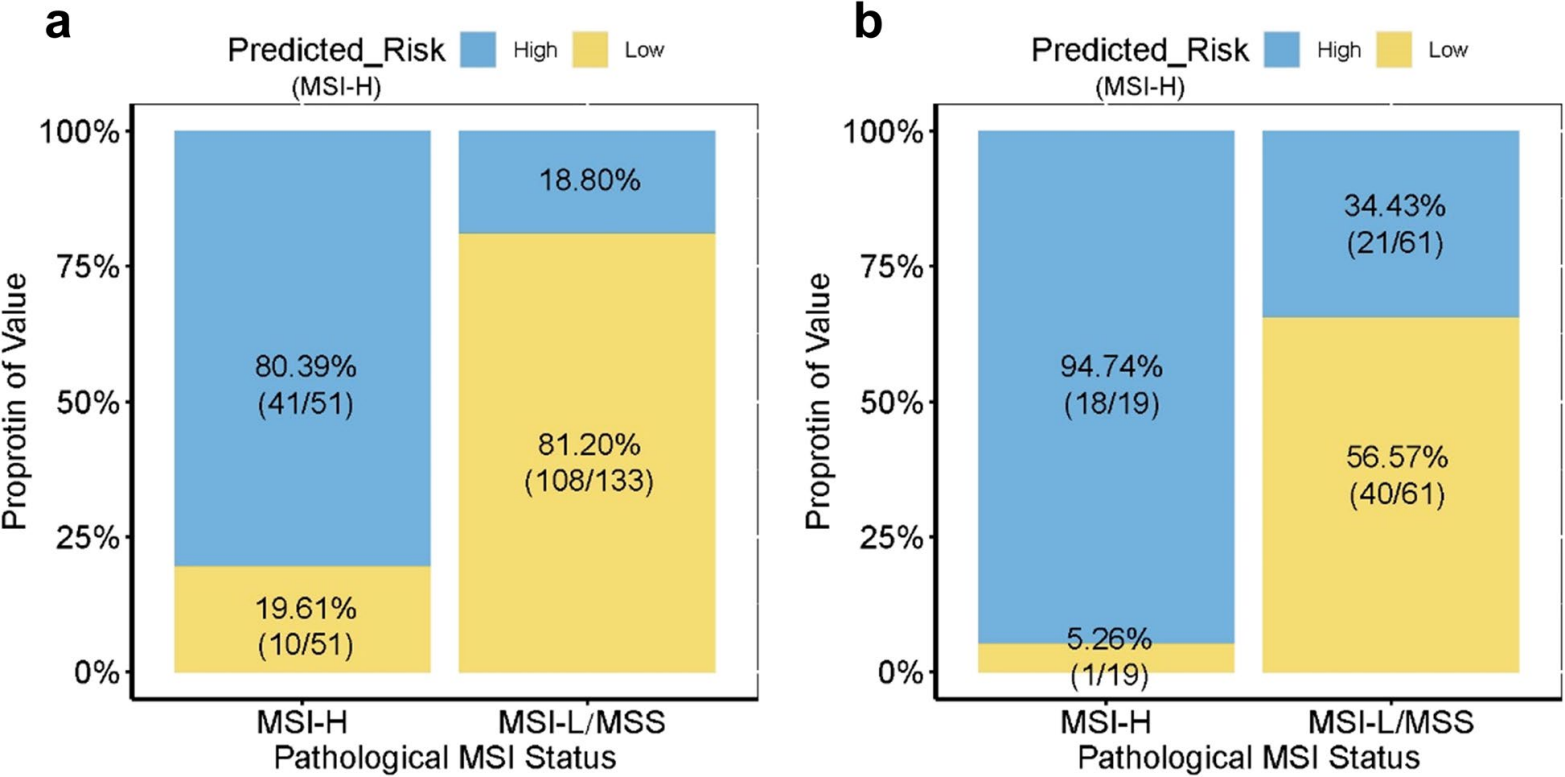
Bar chart of discrimination performance for microsatellite instability status of the combined model. The bar chart demonstrated the result of combined model for differentiating microsatellite instability status of gastric cancer in the training set (**a**) and the validation set (**b**). The blue box showed the predicted microsatellite instability high (pred-MSI-H), and the yellow box showed the predicted microsatellite instability low or microsatellite stable (pred-MSI-L/MSS)

The application of the combined model and nomogram is shown in Figs. 3 and 4. Patients were categorized into predicted MSI-H (pred-MSI-H, RS >  − 0.81) or MSI-L/MSS (pred- MSI-L/MSS, RS ≤  − 0.81) groups based on the RS threshold from ROC analysis (Fig. 5).

### Prognostic significant of the combined model

**Fig. 6:**
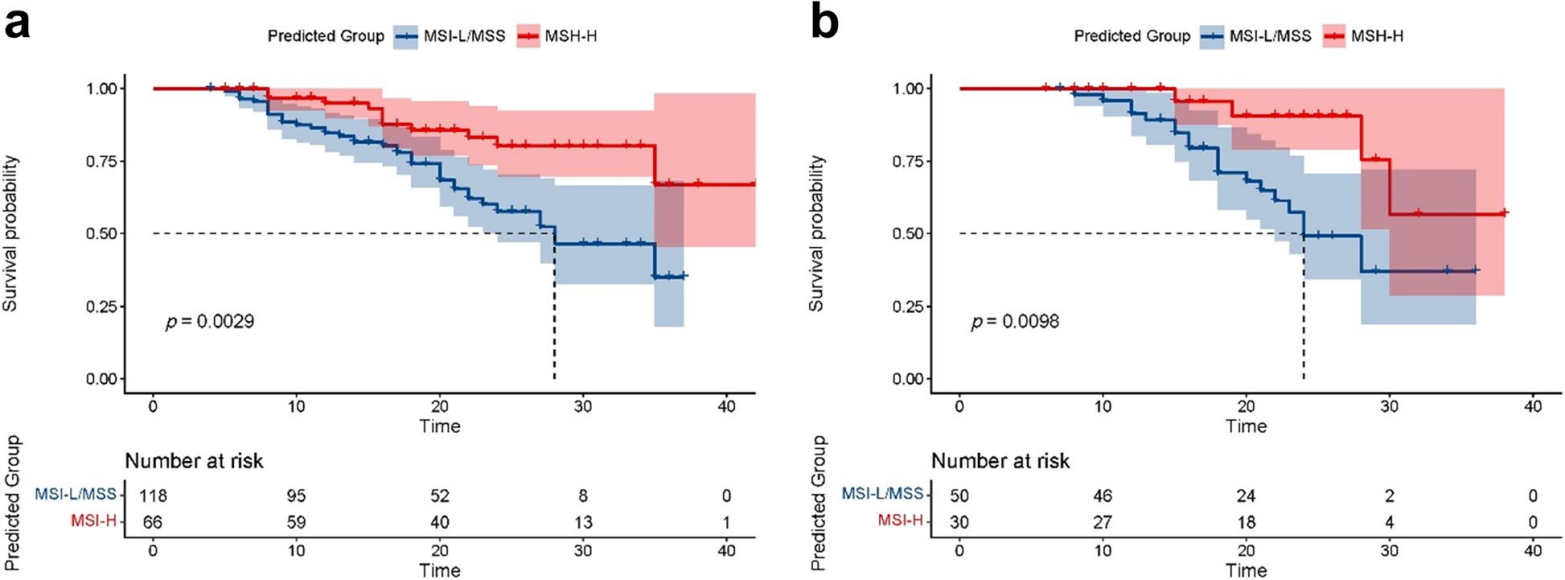
Kaplan–Meier survival curve of progression-free survival (RFS) stratified by the combined model. Survival risk stratification of the combined model was measured by Kaplan–Meier analysis and the *p* value was calculated using log-rank test according to risk scores grouped by the optimal cutoff. The result showed the nomogram had significant capability to risk stratification (*p* < 0.05), both in the training set (**a**) and the validation set (**b**). The red line was the patients with predicted microsatellite instability high (pred-MSI-H), and the blue line showed the patients with predicted microsatellite instability low or microsatellite stable (pred-MSI-L/MSS)

The median follow-up time for the 264 patients in this study was 20.0 months (range 4.0–42.0 months), with 73/264 (27.7%) cases experiencing tumor recurrence. Kaplan–Meier survival analysis showed that the median RFS for the entire group was 35 months and the 2-year RFS rate was 65.6%. In the training set, the median RFS for pred-MSI-H was over 42 months and the 2-year RFS rate was 80.3%; the median RFS for pred-MSI-L/MSS was 28 months and the 2-year RFS rate was 57.6%, with a significant difference between the two groups (log-rank, *p* = 0.003). In the validation set, the median RFS for pred-MSI-H was over 38 months and the 2-year RFS rate was 90.6%; the median RFS for pred-MSI-L/MSS was 24 months and the 2-year RFS rate was 49.1%, with a significant difference between the two groups (log-rank, *p* = 0.010) (Fig. 6).

## Discussion

The results of this study showed that a combined model based on DLCT quantitative parameters and clinico-radiologic features could predict the MSI status of GC preoperatively with satisfactory predictive performance. The AUC value, sensitivity, specificity, and accuracy in the validation set were 0.879, 78.95%, 75.40%, and 76.25%, respectively. In addition, the risk stratification based on the combined model showed a potential prognostic significance after gastrectomy.

In this study, tumor location and CT_N staging were identified as independent predictors of MSI status in GC. MSI-H GC was more frequently found in the antrum and pylorus, consistent with previous research findings [16]. This implies that the mechanism of tumorigenesis in different regions might be distinct, resulting in different gene mutations and biological phenotypes [26]. The MSI-H group exhibited earlier staging than the MSI-L/MSS group. However, multivariate logistic regression showed that only CT_N staging was an independent predictor, indicating that there might be a higher association between MSI status and lymph node metastases. In addition, we found that the age of MSI-H GC was higher than that of MSI-L/MSS GC, possibly because of the accumulation of *hMLH1* gene promoter methylation with age [7]. The proportion of Lauren intestinal type GC was higher in the MSI-H group than in the MSI-L/MSS group, which was consistent with previous literature reports and might be one reason for the good prognosis in MSI-H GC [27].

We found that all the DLCT parameters in VP but not AP showed significant differences. This might be related to the enhancement pattern of gastric cancer [28]. Previous studies reported that most GC showed peak enhancement in the venous phase or delayed phase [28–31], affecting the iodine uptake. Tsurumaru et al. speculated that intratumoral fibrosis, tumor cell infiltration patterns, and lack of neovascularity might contribute to the marked contrast enhancement in VP [30, 31], which might result in an obvious difference in CT attenuation and DLCT parameters in VP between different types of GC. DLCT has been successfully used to detect iodine concentration in vitro [32] and reflect quantified tissue blood flow in vivo [33]. NIC may reduce variations caused by individual differences in hemodynamics between individuals, thereby more accurately reflecting tissue blood supply in the tumor [20]. According to pathological studies [34–36], MSI-H GC could be distinguished from MSI-L/MSS GC by having a lower microvessel count, less angiogenesis-related gene expression, more tumor necrosis, and a greater mucin content. This might cause the lower ID_VP_ and NID_VP_ values due to poor blood supply of MSI-H GC. Zeff represents the average atomic number of the constituent elements in the tissue, which is related to tissue density and iodine concentration. λHU reflects the degree of X-ray attenuation at different energy levels, which is another important parameter reflecting tissue characteristics [17, 18]. We found that Zeff and λHU values differed significantly between different MSI-H GC and MSI-L/MSS GC, suggesting that the two groups might differ in tissue density, cell type, and material composition. Previous studies had revealed that MSI-H GC had a larger tumor mutation burden, increased tumor necrosis, a mucous component, and lymphocyte infiltration [36, 37], which might explain the differences in material composition between the two groups of GS.

In this study, we constructed a combined model to predict the MSI status of gastric cancer using the combined DLCT parameter C_DLCT_ and clinico-radiologic features and visualized it in a nomogram to facilitate clinical application. An independent validation set was employed to validate the model. The combined nomogram showed satisfactory predictive performance with an AUC of 0.879. To investigate the clinical application value of the combined prediction model, we also explored the relationship between predicted MSI status and the prognosis of patients after surgery. Kaplan–Meier survival analysis showed that the prediction result could stratify the prognosis in terms of RFS. MSI-H GC might have more immune cell infiltration, induce a stronger anti-tumor immune response, thus prevent tumor invasion, and induce tumor cell apoptosis, finally resulting in a better prognosis [6, 7, 38]. The combined model could serve as an important indicator for recurrence risk stratification of GC and provide a basis for choosing personalized treatment strategy. MSI-H high-risk patients might benefit from immunotherapy, while MSI-L/MSS high-risk patients might have a poor prognosis, require intensive treatment to prevent tumor recurrence, and benefit from surgery combined with adjuvant chemotherapy [6].

In addition, ID, NID, Zeff, and λHU could also be obtained using other dual-energy CT imaging technologies, such as dual-source and rapid voltage-switching systems. However, further research is required to investigate whether the findings of this study can be applied to other dual-energy CT scanners since different technologies have different strategies of high- and low-energy separation.

There are also some limitations in the present study. First, this was a single-center study, so multi-center validation was needed for widely application of the prediction model. Second, the patients enrolled in this study were locally advanced GC and did not include early gastric cancer, which might introduce certain selection bias. This is because the lesion of early gastric cancer might be too small to be detected on CT images. Third, the manual ROI drawing might introduce some subjectivity in terms of the delineation of tumor boundaries. Fourth, a delayed phase scan was not routinely performed for suspected gastric cancer in our institution, although several studies showed some types of GC exhibited gradual and delayed enhancement [30, 31]. Fifth, since the follow-up time is short with a median follow-up time of 35.0 months, no analysis of overall survival has been performed. Further research is needed to solve the above limitations.

In conclusion, the present study demonstrated that the combined prediction model based on DLCT parameters, tumor location, and CT_N staging provides a noninvasive and practical tool for preoperative evaluation of the MSI status of GC. The predicted MSI status can stratify the risk of recurrence after radical gastrectomy. This may assist clinicians in determining individualized treatment plan, assessing the risk of recurrence after surgery in clinical practice, and improving patient prognosis.

### Supplementary Information

**Additional file 1: Text S1.** DLCT Examination. **Table S1.** Inter-observer reproducibility for quantitative parameters measurement. **Table S2.** Inter-observer agreement for radiological characteristics. **Fig. S1.** Calibration curves and decision curve analysis (DCA) of the prediction model.

## Data Availability

The datasets used and/or analyzed during the current study are available from the corresponding author on reasonable request.

## Abbreviations

AUC: Area under the curve
C_DLCT_: Combined DLCT parameter
DLCT: Dual-layer spectral detector CT
DCA: Decision curve analysis
GC: Gastric cancer
ICC: Intraclass correlation coefficient
ID: Iodine density
λHU: The slope of the spectral curve
MD: Maximum diameter
MMR: Mismatch repair
MSI: Microsatellite instability
MSI-H: Microsatellite instability high
MSI-L: Microsatellite instability low
MSS: Microsatellite stable
MT: Maximum thickness
NID: Normalized iodine density
pred-MSI-H: Predicted MSI-H
pred-MSI-L/MSS: Predicted MSI-L/MSS
ROC: Receiving operating curve
ROI: Region of interest
RS: Risk score
VMI: Virtual monoenergetic images
Zeff: Effective atomic number
