# Supplementary material for: Dual-layer spectral-detector CT for predicting microsatellite instability status and prognosis in locally advanced gastric cancer
Source: Insights Imaging. 2023 Sep 19;14:151. doi: 10.1186/s13244-023-01490-x (PMC10509117; doi:10.1186/s13244-023-01490-x)
Supplement: Supplementary file 1 — Additional file 1: Text S1. DLCT Examination. Table S1. Inter-observer reproducibility for quantitative parameters measurement. Table S2. Inter-observer agreement for radiological characteristics. Fig. S1. Calibration curves and decision curve analysis (DCA) of the prediction model. [file 13244_2023_1490_MOESM1_ESM.pdf]

Dual-layer spectral-detector CT for predicting microsatellite instability status and prognosis in locally advanced gastric cancer

ELECTRONIC SUPPLEMENTARY MATERIAL

Text S1 DLCT Examination

Patients fasted for 6-8 hours before the examination. Then, 10 mg of scopolamine hydrochloride (1 mL; 10mg; Hangzhou Minsheng Pharmaceutical Group Co., Ltd., China) was administrated intramuscularly 10–15 minutes before CT examination to reduce gastrointestinal motility artifacts, followed by drinking 800–1000 ml of warm water. Patients were examined in a craniocaudal direction and supine position during breath-hold. After the nonenhanced scan was performed, a standard bolus dosage of 1.5 mL/kg nonionic contrast agent (iopromide 370 mg I/mL, Ultravist 370, Bayer Schering Pharma, Germany) was injected at a rate of 3.0 mL/s via the cubital vein through a pump injector (Optivantage, Mallinckrodt, USA), followed by 40 mL saline flush at the same rate. The arterial phase (AP) and venous phase (VP) images were captured at a delay of 15 s and 50 s after the descending aorta reached the threshold of 120 Hounsfield units (HU) by using a bolus-tracking technique. The acquisition parameters were as follows: tube voltage, 120 kVp; helical pitch, 1.203; gantry rotation time, 0.5 s; matrix, 512 × 512. Tube current modulation was enabled with a 3D DoseRight Index (Philips Healthcare) of 22.

Conventional CT images were reconstructed with an iterative hybrid reconstruction algorithm (iDose^4, level 3, Philips Healthcare) with a standard soft tissue kernel (B). Spectral base images were reconstructed via a dedicated spectral reconstruction algorithm (Spectral, level 3, Kernel B, Philips Healthcare) to generate all spectral parameters. All axial images were reconstructed at 1.0 mm slice thickness with a 1.0 mm section increment.

Table S1 Inter-observer reproducibility for quantitative parameters measurement

Parameter	Inter-observer reproducibility
	ICC (95% CI)
**MD, cm**	0.867 (0.821, 0.884)
**MT, cm**	0.893 (0.862, 0.914)
**Arterial phase**	
CT_{40keV_AP}, HU	0.921 (0.896, 0.948)
CT_{70keV_AP}, HU	0.933 (0.905, 0.956)
ID_{AP}, mg/ml	0.943 (0.922, 0.969)
NID_{AP}	0.902 (0.886, 0.927)
$Zeff_{AP}$	0.915 (0.894, 0.935)
$λHU_{AP}$	0.886 (0.8564, 0.902)
**Venous phase**	
CT_{40keV_VP}, HU	0.932 (0.915, 0.956)
CT_{70keV_VP}, HU	0.926 (0.904, 0.947)
ID_{VP}, mg/ml	0.946 (0.925, 0.969)
NID_{VP}	0.913 (0.894, 0.940)
$Zeff_{VP}$	0.934 (0.902, 0.965)
$λHU_{VP}$	0.872 (0.851, 0.893)

Data are expressed as intraclass correlation coefficient with 95%CIs in parentheses. *A* hepatic arterial phase; *ICC* intraclass correlation coefficient; *ID* iodine density; *λHU* the slope of spectral HU curve; NID, normalized iodine density; *V* venous phase; *Zeff* effective atomic number.

Table S2 Inter-observer agreement for radiological characteristics

**Features**	**Reader 1**	**Reader 2**	**Disagreemen t**	**Kappa (95 % CI)** *
Tumor location				0.970 (0.948, 0.992)
Cardia/Fundus	77 (29.2)	78 (29.5)	5 (6.3)	
Corpus	90 (34.1)	91 (34.5)	7 (7.4)	
Antrum/Pylorus	97 (36.7)	95 (36.0)	2 (2.1)	
CT_T Staging				0.787 (0.754, 0.820)
T2	54 (20.5)	53 (20.1)	11 (18.6)	
T3	101 (38.3)	99 (37.5)	36 (30.5)	
T4a	109 (41.3)	112 (42.4)	25 (20.3)	
CT_N Staging				0.871 (0.846, 0.896)
N0	36 (13.6)	35 (13.3)	11 (26.8)	
N1	82 (31.1)	82 (31.1)	12 (13.6)	
N2	103 (39.0)	102 (38.6)	15 (13.6)	
N3	43 (16.3)	45 (17.0)	10 (20.4)	

Except where indicated otherwise, data are expressed as numbers with percentages in parentheses.

* Data are expressed as kappa coefficient with 95% CIs in parentheses.

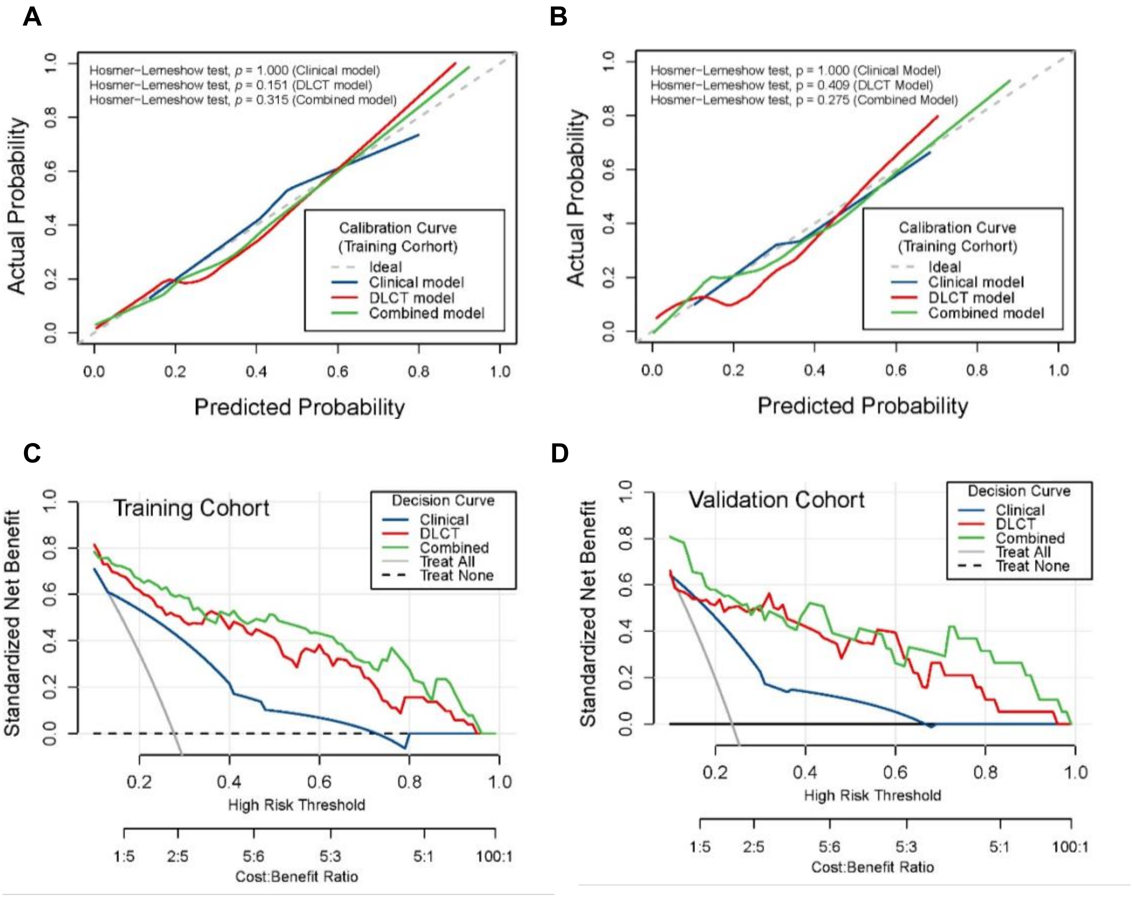

Fig. S1 Calibration curves and decision curve analysis (DCA) of the prediction model. Calibration curves in the training (**A**) and validation (**B**) sets, respectively. The calibration curves display calibration of the nomogram based on the agreement between the forecasted risk of microsatellite instability and final pathological results. The Hosmer–Lemeshow test was not significant for all models (all $p > 0.05$), demonstrating a good fit. DCA for the different models in training (**C**) and validation (**D**) sets, respectively. The area under the decision curve of the models showed the clinical utility of the corresponding strategies, which were better than the "treat all" (gray) or "treat none" (black) strategies, indicating the good performance of the models in terms of clinical application, with the combined model exhibiting the best.
